# The Value of ^18^F-FDG PET/CT and Abdominal PET/MRI as a One-Stop Protocol in Patients With Potentially Resectable Colorectal Liver Metastases

**DOI:** 10.3389/fonc.2021.714948

**Published:** 2021-11-09

**Authors:** Nina Zhou, Xiaoyi Guo, Hongwei Sun, Boqi Yu, Hua Zhu, Nan Li, Zhi Yang

**Affiliations:** ^1^ Key Laboratory of Carcinogenesis and Translational Research (Ministry of Education/Beijing), NMPA Key Laboratory for Research and Evaluation of Radiopharmaceuticals (National Medical Products Administration), Department of Nuclear Medicine, Peking University Cancer Hospital and Institute, Beijing, China; ^2^ United Imaging Research Institute of Intelligent Imaging, Beijing, China

**Keywords:** colorectal cancer, liver metastasis, PET/CT, PET/MRI, clinical impact analysis

## Abstract

**Purpose:**

The aim of this study was to evaluate the clinical value of simultaneous positron emission tomography/computed tomography (PET/CT) and abdominal positron emission tomography/magnet resonance imaging (PET/MRI) in the detection of liver metastases and extrahepatic disease (EHD) in patients with potentially resectable colorectal liver metastases (CLM).

**Methods:**

Fifty-six patients with CLM underwent conventional imaging (chest and abdomen CT, liver contrast-enhanced CT or MRI) and PET imaging [fluorine-18 fluorodeoxyglucose (^18^F-FDG) PET/CT and subsequent liver PET/MRI] for staging or restaging. Diagnostic ability of PET imaging was compared with conventional imaging. Abnormal findings were correlated with follow-up imaging and/or histology. The influence of the PET imaging findings was categorized for each patient in relation to operability and other significant findings. The clinical management included three modalities (surgery for resectable CLM, unresectable CLM with conversion treatment, and systemic therapy). The clinical impact of the imaging modality was analyzed. The operative histopathological analysis and/or imaging follow-up were performed as the standard of reference.

**Results:**

This study enrolled a total of 56 patients (median age 60 years, 62.5% were male, 36 with colon cancer and 20 with rectal cancer). For EHD detection, PET/CT detected more EHD than conventional imaging (60.7% *vs.* 46.4%). PET/CT had different findings in 19 (33.9%) patients, including downstaging in 4 (7.1%) patients and upstaging in 15 (26.8%) patients. For liver lesion detection, PET/MRI showed comparable detection ability with CE-MRI and CE-CT (99.5%, 99.4%, and 86.5%, respectively) based on lesion analysis, much higher than PET/CT (47.5%). PET imaging had a major impact in 10/56 (17.9%) patients (4 from unresectable to resectable, 6 from resectable to unresectable) and a minor impact in 4/56 (7.1%) patients for changing the surgery extent. The therapeutic strategies had been altered in a total of 14/56 patients (25%) after PET/CT and PET/MRI scans.

**Conclusion:**

The results of this study indicate that simultaneous ^18^F-FDG PET/CT and abdominal PET/MRI scans can provide accurate information regarding CLM status and EHD, and can affect the management of 25% of the patients by changing the therapeutic strategies determined by conventional imaging. This new modality may serve as a new one-stop method in patients with potentially resectable CLM.

## Introduction

Colorectal cancer (CRC) is one of the most common cancers. Long-term patient outcome is heavily influenced by the initial stage. Approximately 25%–30% of patients have hepatic metastases at presentation. Recurrent disease is seen in up to 30% of patients within 2 years of initial resection, in which the majority manifests as liver metastases (1). Liver resection for colorectal liver metastasis (CLM) is an established treatment and offers a realistic chance of disease-free survival. Five-year survival rates following liver resection have been reported at approximately 30% with a perioperative mortality rate of <3% (2–4). Therefore, identifying the group of patients who would benefit from a liver resection for CLM is paramount. In addition, approximately 20%–30% of newly diagnosed patients with CRC present with synchronous metastases. Some extrahepatic disease no longer represents an absolute contraindication to surgery, but necessitates a wider operative field or second operation, if technically feasible, allowing a truly curative surgical resection (5–8).

Although practice varies between treatment centers, many lines of evidence suggest that the best method for detection of liver metastases from CRC are computed tomography (CT) and magnetic resonance imaging (MRI) (9). For lesions with a diameter of less than 10 mm, MRI is a more sensitive modality than CT (10, 11), and specifically in hepatobiliary MRI with specific contrast enhancers (such as gadoxetate), showing a higher accuracy of lesion detection (12–15). Many studies have investigated the optimal modality for imaging hepatic metastases, finding pooled sensitivity on a per-lesion basis of 88% for MRI, 74% for CT, and 79% for positron emission tomography/computed tomography (PET/CT) (9, 16–18).

For the detection of extrahepatic metastases (EHD) and local recurrence at the site of the initial colorectal surgery, CT and PET/CT scans are used (19). A prospective randomized trial evaluating high-quality CT and PET imaging involving 263 patients showed only a 7.6% change in management following PET (20, 21), while a retrospective analysis reported, in one-third (33.3%) of patients, a change in intended curative therapy to palliative therapy, or *vice versa* (22).

The soft-tissue contrast provided by CT in a PET/CT scan may not be sufficient enough for small lesion detection in the clinical practice. Hence, PET and MRI hybrid units [positron emission tomography/magnet resonance imaging (PET/MRI)] were initially manufactured in 2011 and were soon approved in both the United States and the European Union. Until now, the evidence on the potential value and role of PET/MRI in clinical medicine is still accumulating (23–25). Evaluation of small liver lesions can be challenging using PET/CT due to low image contrast. As a potential remedy, PET/MRI may be used to detect small liver lesions with its high soft-tissue contrast and functional diffusion-weighted imaging sequence (23, 26, 27).

The aim of this study was to evaluate the influence of ^18^F-FDG PET/CT in the detection of EHD and diagnostic efficiency of PET/MRI in the detection of intrahepatic lesions in patients with potentially resectable CLM.

## Materials and Methods

### Patient Enrollment

**Figure 1:**
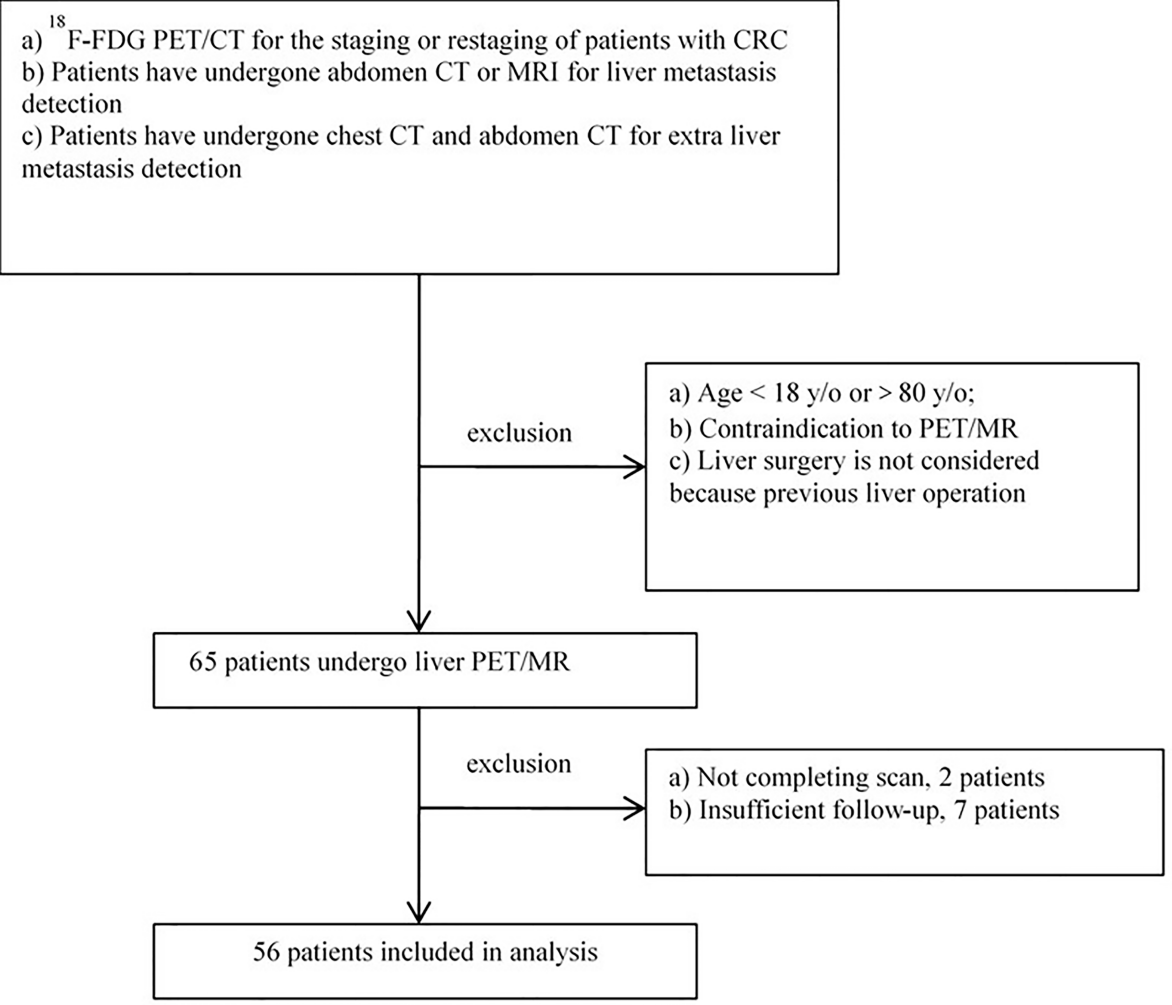
The schematic of enrollment and exclusion criteria.

This study was performed under a single-center prospective imaging protocol and was approved by the Medical Ethics Committee of Peking University Cancer Hospital (ethical approval no. 2018KT110-GZ01). Between October 2019 and December 2020, patients referred to the hepatobiliary multidisciplinary team for consideration of liver resection for CLM after initial imaging (including CT of chest and either contrast-enhanced CT or MRI of abdomen) were enrolled. These candidates took an ^18^F-FDG PET/CT scan first, followed by a delayed abdomen PET/MRI scan. All patients were provided with written informed consent before study participation. Inclusion criteria for the study participation include any of the following conditions (**Figure 1**):

(a) ^18^F-FDG PET/CT for the staging or restaging of patients with colorectal cancer.
(b) Patients have undergone abdomen contrast-enhanced (CE) CT or MRI for liver metastasis detection, and the interval time between PET and CT/MRI was less than 30 days.
(c) If there were positive or indeterminate findings on PET/CT, these must have confirmatory evidence with either histology or follow-up imaging at least 3 months.

Additionally, patients with any of the following conditions have been excluded:

(a) Age <18 years or >80 years because of the ethical restriction;
(b) Contraindication to PET/MRI imaging;
(c) Insufficient imaging follow-up to confirm the metastasis lesion, or insufficient follow-up for clinical therapy.

### Imaging

Imaging was performed using a PET/CT scanner (Biograph64, SIEMENS, Erlangen, Germany) operated in 3D Flow Motion (bed entry speed 1 mm/s) from the apex of the skull to the mid-thigh, with a PET axial field of view of 21.6 cm. The PET images were reconstructed by the TrueX + TOF method offered by the vendor. The CT component was a 64 slice spiral CT, and low-dose CT scans without contrast enhancement were acquired in CARE Dose 4D mode (120 kV, 3.0 mm slice thickness). The patients were instructed to fast for at least 6 h before ^18^F-FDG injection. In all cases, the serum glucose concentration met our institutional requirement (≤140 mg/dl). The injected activity was 3.7 MBq/kg, and the time from injection to scan was 60 min. The PET/CT scan lasted about 15 min.

^18^F-FDG PET/MRI was performed on an integrated 3.0-T Time-of-flight PET/MRI scanner (uPMRI790, UIH, Shanghai, China). The scan started at 120–180 min after ^18^F-FDG administration. The time interval between ^18^F-FDG PET/CT scan and PET/MRI scan were 60–90 min in order to get a delay liver PET. Each patient underwent the same protocol as described in the following. Body array coil was placed around the individual and covered the entire liver. Respiratory gating was used in MRI acquisition whenever possible. PET reconstruction was conducted using a 3D-Ordered Subsets Expectation Maximization (3D-OSEM) algorithm applied on a 256 × 256 matrix. A four-compartment-model attenuation map (μ-map) automatically generated based on a water-fat-imaging MRI sequence was used for PET attenuation correction. The PET images were smoothed by a Gaussian filter with 3 mm full width at half maximum (FWHM). The MRI sequences were performed simultaneously with PET acquisition, including T2WI with fat saturation, T1WI, and DWI. The mean scan time for PET/MRI was 20 ± 6 min. The detailed MRI parameters are shown in **Supplementary Table 1**.

### Image Analysis

Images were reviewed using our local picture archiving and communication system (PACS), by two accredited radiologists with more than 4 years of experience in hybrid PET/CT and PET/MRI imaging. In PET/CT imaging, lesions were rated as metastases when PET had positive uptake foci with abnormal density on CT. In PET/MRI imaging, liver lesions were rated as metastases when at least two of the three following criteria were met: (a) hyperintense on T2WI, (b) diffusion restriction on DWI, and (c) PET-positive. Moreover, CE-CT and CE-MRI images from PACS were reviewed by two radiologists. All liver lesions detected on PET/CT and PET/MRI were documented for patients with less than 10 lesions. Each patient was categorized into negative or positive of extrahepatic disease on PET/CT. The number/extent of liver metastasis on PET/MRI was also compared with CE-CT or CE-MRI scans.

### Analysis of Clinical Impact

**Figure 2:**
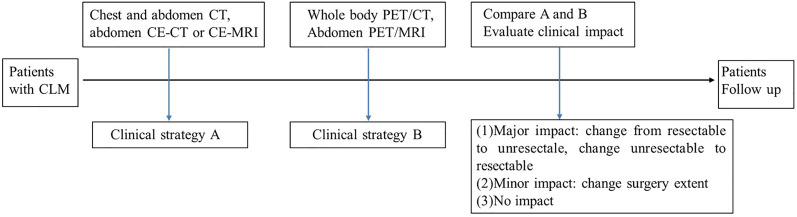
The analysis flow chart of clinical impact.

The conventional imaging (chest/abdomen CT and liver MRI/CT) and PET imaging (PET/CT and liver PET/MRI) were assessed by a multidisciplinary team (MDT), and then the patients were organized into three groups including (a) resectable CLM, (b) unresectable CLM with “conversion” as a strategic treatment goal, and (c) unresectable CLM with systematic therapy. They were further categorized as to whether the PET imaging findings had a major impact (change resectable to unresectable, change unresectable to resectable), minor impact (change resectable extent), or no impact (no therapy changed). For the patients who received liver surgery, the lesion number identified on imaging was compared with the final resection lesion number. The clinical treatment, biopsy, surgical pathologic analysis, correlation with prior imaging findings, and clinical and imaging follow-up were used as the reference standard for the image findings. The follow-up was conducted at least 90 days after the initial PET/CT and PET/MRI study (**Figure 2**).

## Results

### Patient Characteristics

Out of the 65 patients initially enrolled, 2 were excluded for an incomplete delayed PET/MRI scan, and another 7 were excluded for insufficient follow-up. Thus, 56 patients with colorectal liver metastases were included in the final analysis. Their median age was 59 years (31–80 years), where 36 patients had colon cancer and 20 patients had rectal cancer, with 21 patients receiving chemotherapy within 3 months. According to the reference standard and follow-up, a total of 206 liver metastatic lesions were detected, including 162 lesions with diameter less than or equal to 10 mm (range 2–10, mean 6.5 ± 1.9), and 44 lesions with diameter larger than 10 mm (range 11–70, mean 19.9 ± 12.8). Clinical follow-up showed that 26 patients received surgery, 4 patients receives surgery after conversion treatment, and 26 patients received systematic therapy. The patient characteristics of this study are summarized in **Table 1**.

**Table 1:** Patient characteristics of this study.

Patients Characteristic	Number
Age (years)	31–80 (median 60)
Male/Female	35/21
Primary tumor	
Colon cancer	36
Rectal cancer	20
Pathology subtypes	
Moderate differentiated adenocarcinoma	50
Poorly differentiated adenocarcinoma	4
Mucinous adenocarcinoma	2
Staging/Restaging	7/49
Chemotherapy within 3 months (Yes/No)	21/35
Conventional Imaging for CLM	
CE-CT	12
CE-MRI	44
Clinical follow-up	
Surgery	26
Surgery after conversion treatment	4
Systematic therapy	26

### PET/CT Imaging for Extrahepatic Disease Detection

PET/CT detected EHD in 34 patients, 14 of which have oligometastatic disease (OMD). The conventional CT detected EHD in 26 patients.

Based on patient analysis, PET/CT had different findings in 19 patients, including downstaging in 4 patients and upstaging in 15 patients.

**Figure 3:**
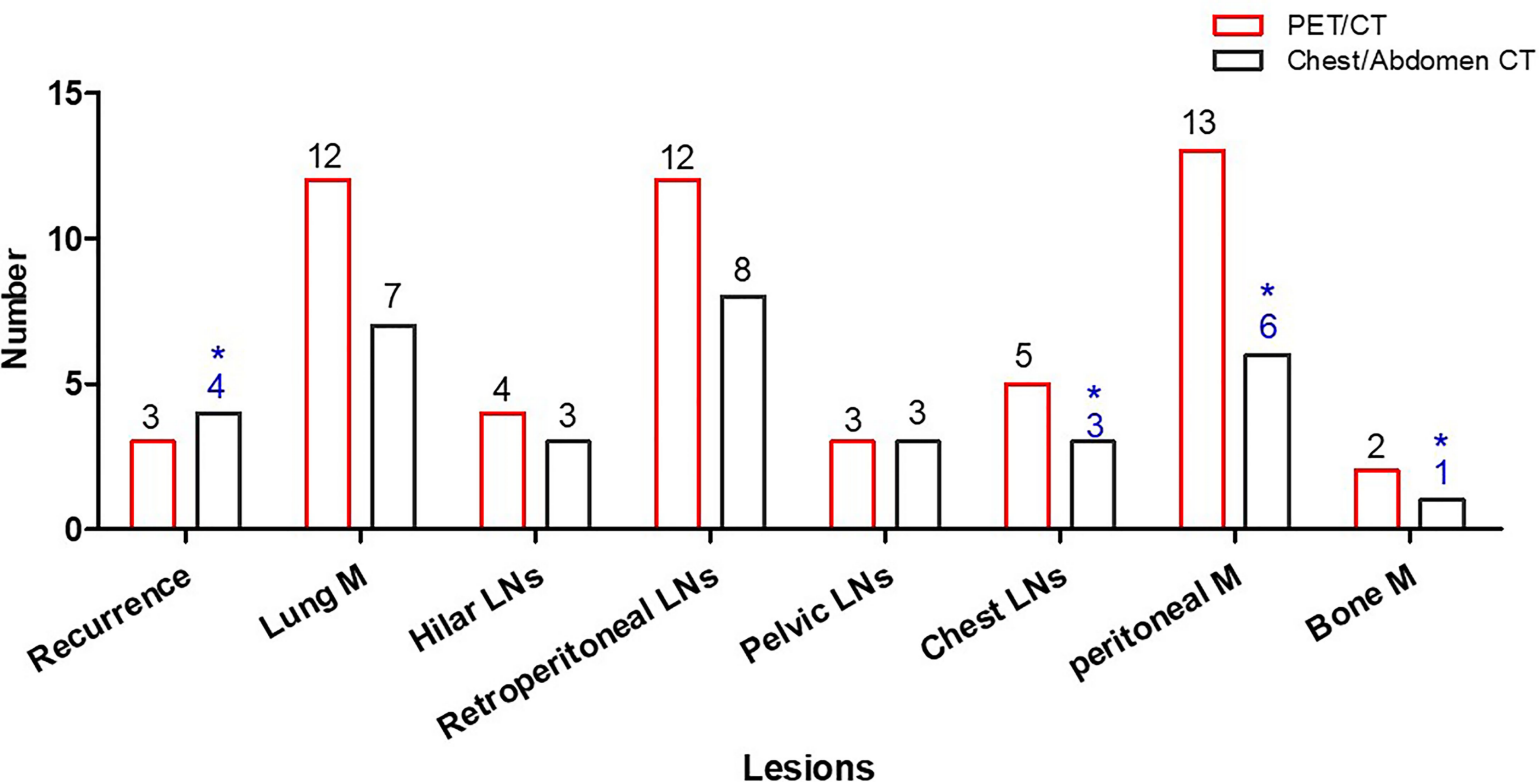
Patient number with EHD detected by PET/CT and common CT. Common CT had false-positive results in four patients (*).

Based on lesion analysis, PET/CT detected more EHD for hilar lymph nodes (hilar LN) (4 *vs*. 3), lung metastases (lung M) (12 *vs*. 7), retroperitoneal lymph nodes (retroperitoneal LNs) (12 *vs*. 8), chest lymph nodes (chest LNs) (5 *vs.* 2), peritoneal nodules (13 *vs*. 6), and bone metastases (bone M) (2 *vs*. 0). PET/CT detected the same iliac lymph nodes, inguinal lymph nodes, and locoregional recurrent. PET/CT ruled out false-positive EHD, including one recurrence, one peritoneal nodule, one mediastinal lymph node, and one bone metastasis (**Figure 3**).

### Diagnostic Efficiency of PET/MRI for Liver Lesions

CE-MRI was performed in 43 patients and CE-CT was performed in 13 patients for evaluation of liver lesion.

Based on patient analysis, PET/CT showed no metastasis in 16 out of 56 metastatic patients, while the other three image modalities (PET/MRI, CE-CT, and CE-MRI) detected liver lesions in all 56 patients. For one patient, CE-CT misdiagnosed the liver lesion as metastasis while PET/MRI showed a mass with restriction on DWI and low ^18^F-FDG uptake on PET/CT. Then, a second primary tumor was diagnosed for the inconsistency with the high ^18^F-FDG uptake of primary lesion, which also confirmed a second primary hepatic cell carcinoma (HCC) by pathology. PET/CT missed one or more lesions in 33 patients, regardless of whether the patients received therapy within 3 months or not (*χ* ^2^ = 2.17, *p* = 0.141).

**Figure 4:**
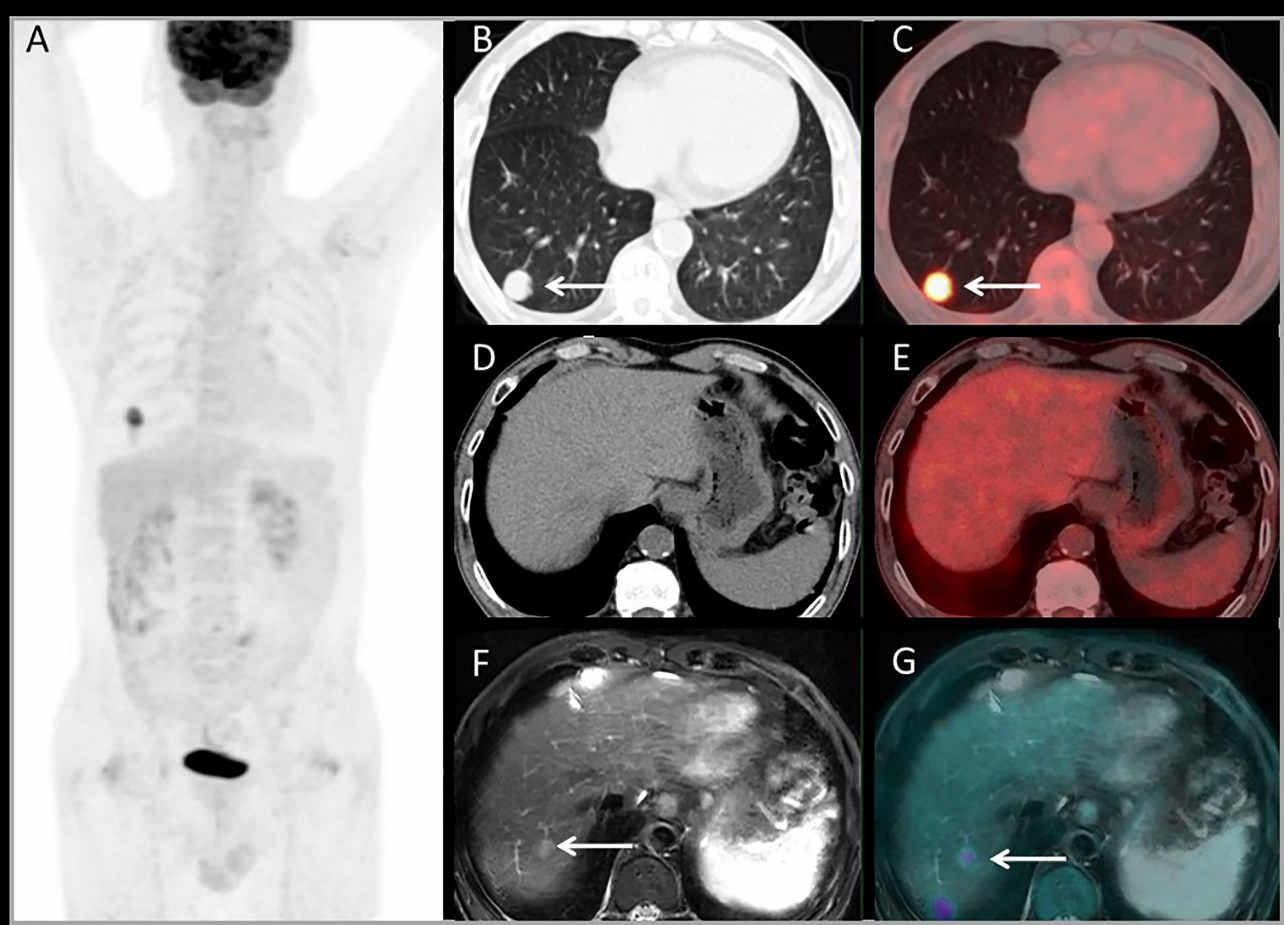
Images of a 63-year-old male with colonic liver metastasis. The MIP **(A)** of PET/CT showed a lesion on right lung but no ^18^F-FDG foci on liver. CT and PET/CT fusion images showed single lung metastasis with high ^18^F-FDG uptake **(B, C)** and no lesions were detected on liver **(D, E)**. T2WI and PET/MRI fusion images showed a single lesion on right liver lobe with mild ^18^F-FDG uptake **(F, G)**.

**Figure 4** shows a patient with liver metastases detected by PET/MRI but missed on PET/CT. The PET/CT detected one lung lesion, so the patient received a simultaneous operation of lung and liver metastases.

**Figure 5:**
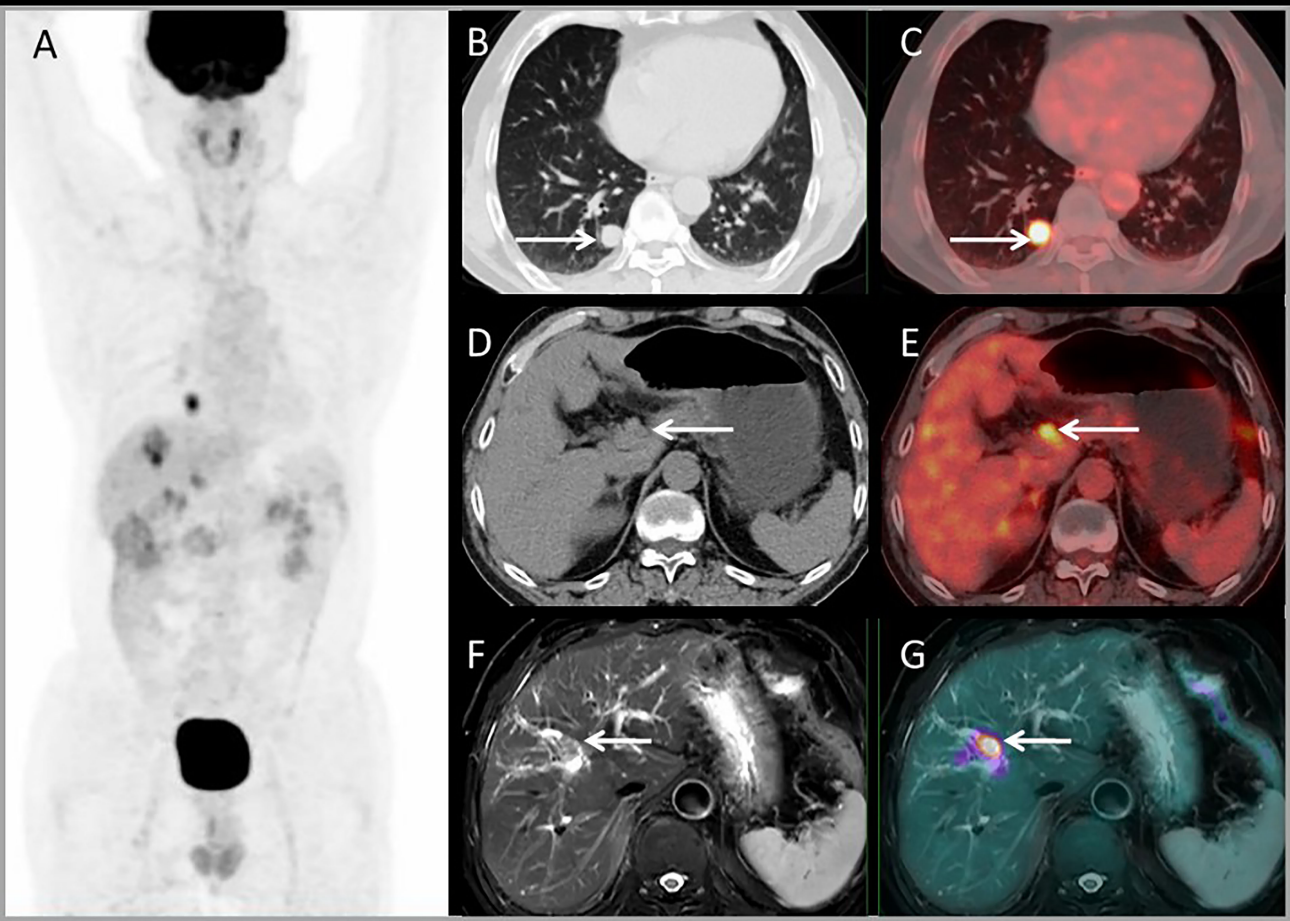
Images of a 64-year-old male with colonic liver metastasis. The MIP **(A)** of PET/CT showed a single lesion on the liver and EHD lesions. CT and PET/CT fusion images showed single lung metastasis **(B, C)** and hilar lymph node metastasis **(D, E)**. T2WI and PET/MR fusion images showed single liver lesion with intrahepatic bile duct infiltration **(F, G)**.

For lesion analysis, there were 206 liver metastases based on standard reference. PET/MRI detected 205/206 (99.5%) metastases, CE-MRI detected 168/169 (99.4%), CE-CT detected 32/37 (86.5%), and PET/CT only detected 98/206 (47.5%). Both CE-MRI and PET/MRI detected three patients with bile duct infiltration (**Figure 5**) and two patients with multiple liver metastases that were unresectable (**Table 2**).

**Table 2:** Diagnostic accuracy of PET/CT and PET/MR compared with MRI/CT for CRLM.

Group	CE-MRI	PET/MR	PET/CT
Patient based (*n* = 43)	43 (100%)	43 (100%)	32 (74.4%)
Lesion based (*n* = 169)	168 (99.4%)	168 (99.4%)	82 (48.5%)
**Group**	**CE-CT**	**PET/MR**	**PET/CT**
Patient based (*n* = 12)*	13 (100%)*	12 (100%)	6 (58.3%)
Lesion based (*n* = 37)	32 (86.5%)	37 (100%)	16 (43.2%)

*One patient with HCC misdiagnosed as metastasis by liver CE-CT while accurately diagnosed by PET/CT and PET/MR.

### The Clinical Impact of PET Imaging

Based on conventional imaging, patients were organized into three groups including (a) resectable CLM (*n* = 32), (b) unresectable CLM with “conversion” as a strategic treatment goal (*n* = 4), and (c) unresectable CLM with systematic therapy (*n* = 20).

Based on PET imaging, patients were organized into three groups including (a) resectable CLM (*n* = 28), (b) unresectable CLM with “conversion” as a strategic treatment goal (*n* = 5), and (c) unresectable CLM with systematic therapy (*n* = 23).

**Figure 6:**
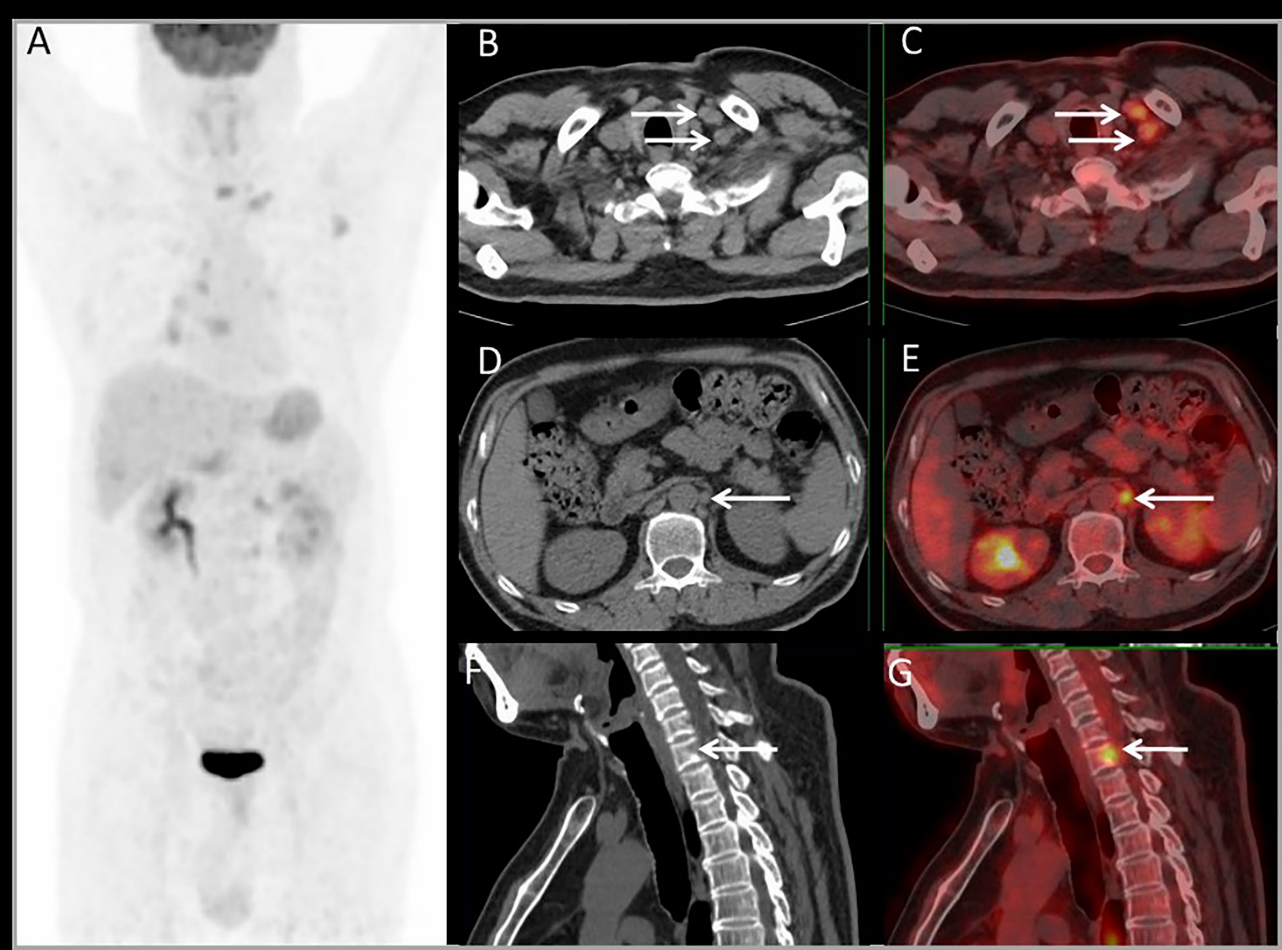
Images of a 54-year-old male with rectal liver metastases. Common CT showed no EHD. The MIP **(A)** of PET/CT showed liver metastasis and multiple EHD. PET and PET/CT fusion images showed left supraclavicular lymph nodes metastases **(B, C)**, retroperitoneal lymph nodes metastases **(D, E)**, and bone metastases on the 7th cervical vertebra **(F, G)**. The therapy strategy was changed from resectable to unresectable (major impact). The patient received systematic therapy after PET/CT restaging.

**Figure 7:**
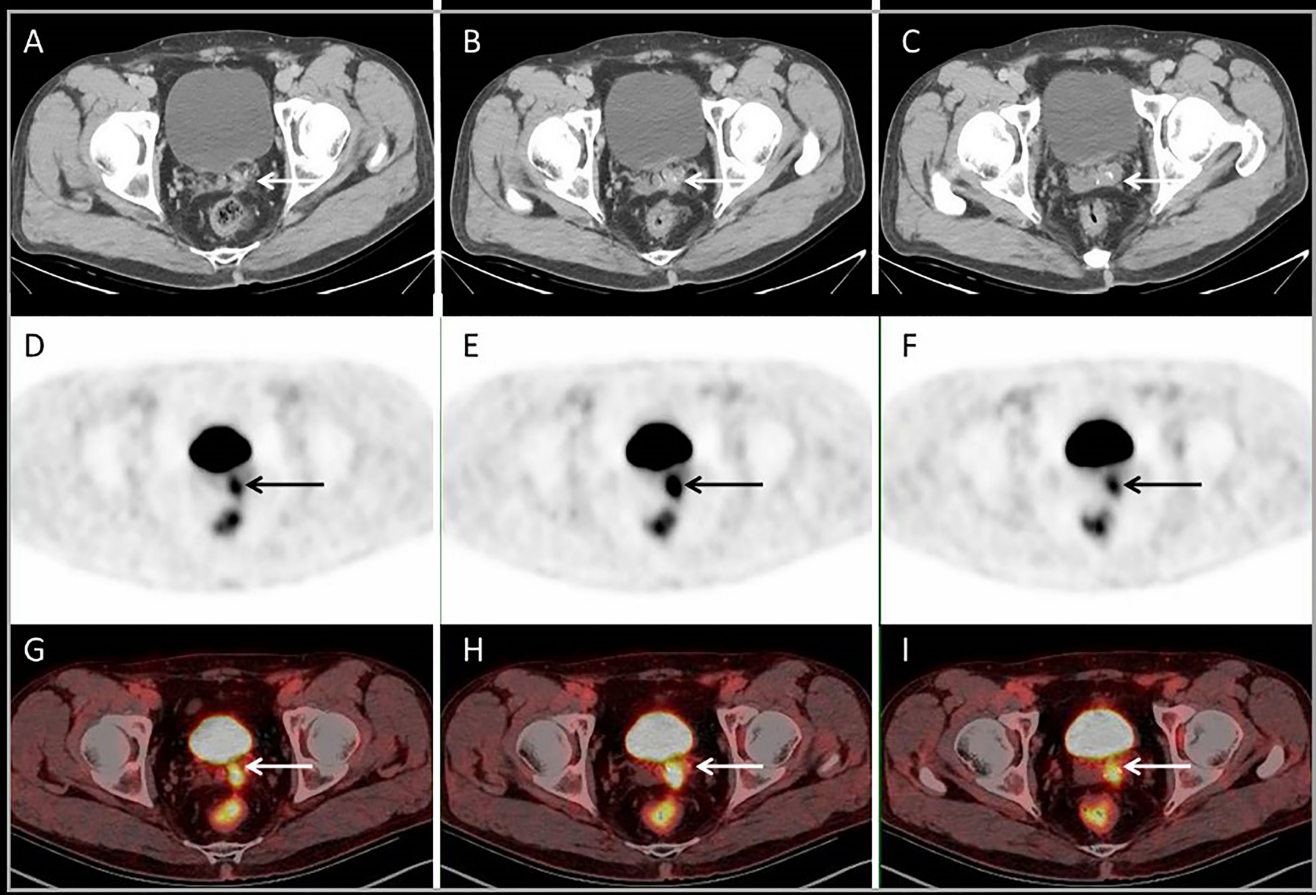
Images of a 52-year-old male with single liver metastasis after surgery of sigmoid cancer resection. Conventional abdomen CT showed no EHD **(A–C)**. PET and PET/CT fusion images showed a peritoneal nodule adjacent to the left seminal vesicle gland **(D–I)**. The surgery extent changed from liver only to combining live and peritoneal nodule (minor impact).

PET imaging had consistent findings with conventional imaging in 37 patients, and PET had different findings in 19 patients. For 19 patients whose PET had different findings, PET downstaged in 4 patients and upstaged in 15 patients (**Table 3**). PET had a major impact in 10 (17.9%) patients by modifying the therapy from unresectable to resectable in 4 patients, and modifying therapy from resectable to unresectable in 6 patients. PET had a minor impact in 4 (7.1%) patients for changing the surgery extent. For the other 5 patients upstaging, there was no impact on their therapy plan. Above all, PET imaging had a clinical impact in 25% of patients. Each patient’s condition and the clinical impact of PET are listed in **Table 3**. Major and minor impact for two typical cases are shown in **Figures 6**, **7**, respectively.

**Table 3:** Additional PET findings in all 19 patients and the clinical impact.

Patients	Convention Imaging Finding	PET Imaging Finding	Therapeutic Comment by Conventional Imaging	Therapy Comment by PET Imaging	Stage	Clinical Impact of PET
1	Recurrence	None	Unresectable	Resectable	Down	Major
2	Mediastinal LN M	Inflammatory LN	Unresectable	Resectable	Down	Major
3	Suspicious right iliac bone M	None	Unresectable	Resectable	Down	Major
4	Peritoneal nodule	None	Unresectable	Resectable	Down	Major
5	Equivocal pelvic bone M	Bone/Peritoneal/retroperitoneal LNs M	Resectable	Unresectable	Up	Major
6	None	Bone/supraclavicular/mediastinal/retroperitoneal LNs M	Resectable	Unresectable	Up	Major
7	None	Recurrent/Peritoneal Nodule M	Resectable	Unresectable	Up	Major
8	None	Peritoneal Nodule M	Resectable	Unresectable	Up	Major
9	None	Retroperitoneal LN	Resectable	Unresectable	Up	Major
10	None	Peritoneal Nodule M	Resectable	Unresectable	Up	Major
11	Equivocal small lung nodule	Lung M/Hilar LN	Resectable	Resectable	Up	Minor
12	No	Hilar LN	Resectable	Resectable	Up	Minor
13	Retroperitoneal LN	Hilar LN/Retroperitoneal LN	Resectable	Resectable	Up	Minor
14	Abdomen Peritoneal Nodule	Abdomen/Pelvic Peritoneal Nodule	Resectable	Resectable	Up	Minor
15	Equivocal small lung nodule	Lung M	Resectable	Resectable	Up	No
16	Equivocal small lung nodule	Lung M	Resectable	Resectable	Up	No
17	Lung inflammatory nodule	Lung M	Resectable	Resectable	Up	No
18	Lung M/Mediastinal LN	Lung M/Mediastinal LN/Recurrent	Unresectable	Unresectable	Up	No
19	Retroperitoneal LN	Retroperitoneal LN/Peritoneal Nodule	Unresectable	Unresectable	Up	No

### Patient Outcome

Based on PET/CT and PET/MRI, patients were organized into three groups including (a) resectable CLM (*n* = 28), (b) unresectable CLM with “conversion” as a strategic treatment goal (*n* = 5), and (c) unresectable CLM with systematic therapy (*n* = 23). Patient’s outcome were as follows.

Group a: For 28 patients with resectable CLM, surgery was performed in 26 patients including liver (*n* = 10), combined liver and lung (*n* = 1), combined liver and upper abdominal nodules (*n* = 3), synchronous colon and liver resection (*n* = 7), metachronous colon then liver resection (*n* = 5). And the other two patients received radiofrequency therapy (n = 2).
Group b: For five patients with “conversion” as a strategic treatment goal, all patients received systemic therapy. The resectability was evaluated 2 months later after optimal treatment and reevaluated 4 months later. At last, four patients received surgery and one patient received liver radiotherapy. For four patients who received surgery, three received liver lesion resection and one received liver lesion and abdominal nodules resection.
Group c: For 23 patients with unresectable CLM, 5 patients had non-operative liver lesion detected by PET/MRI, and 18 of these patients had non-operative EHD detected by PET/CT. They all received systematic therapy.

## Discussion

The current study investigated the role of PET/CT and abdomen PET/MRI in the preoperative work-up of patients with colorectal liver metastases under consideration for curative resection. The evidence from this study demonstrates that patients were upstaging from operative to non-operative disease (10.7%), downstaging from non-operative to operative (7.1%), and changing operation extent (7.1%).

Reduced sensitivity in the detection of sub-centimeter lesions is an accepted limitation of PET/CT, while small liver metastases could be detected more reliably by CE-MRI or CE-CT than by PET/CT. In the authors’ institution, it is a standard practice to use CE-MRI or CE-CT to depict the extent of hepatic disease and use conventional chest CT and abdomen CT to stage extrahepatic disease prior to potential metastasis surgery; PET-CT is performed when conventional CT results are uncertain. In this study, PET-CT had detected more EHD than conventional imaging (60.7% *vs.* 46.4%). A recently published meta-analysis study evaluating PET and PET/CT in patients with liver metastases reported that PET findings resulted in changes in the management of a mean of 24% of patients, with a mean incidence of PET-based EHD of 32% (22).

The current study shows that PET/MRI, as a new diagnostic modality, is feasible for accurate staging with regard to hepatic metastases. It provides a significantly higher diagnostic accuracy in the detection of liver metastases when compared to PET/CT (99.5% *vs.* 47.5%). Additionally, PET/MRI showed comparable ability with CE-CT (100% *vs.* 86.5%) and CE-MRI (99.4% *vs.* 99.4%) for liver lesions. In an earlier trial, Brendle et al. reported that PET/MRI (MRI/DWI/PET) without contrast enhancement showed a relatively lower sensitivity (71%), specificity (80%), as well as diagnostic accuracy (74%) for liver metastases in colorectal cancer. This was mainly because the data contained a relatively high percentage of mucinous tumors, which is known to be challenging for either DWI or PET evaluation (28). Hybrid PET/MRI with contrast enhancement showed higher accuracy for liver metastases (sensitivity 92%–100%, specificity 97%–100%) (29–31). This was consistent with the current findings despite the different acquisition procedures, as this study did not use contrast agents.

There are some limitations in this study. The patients were superselected with CLM, which is diagnosed by CE-CT or CE-MRI ahead. Therefore, radiologists were not blinded to the patients’ history. Considering the expenditure of time and a questionable added value of PET/MRI, we did not use liver-specific contrast agent. Histopathological confirmation of every detected lesion was not available due to ethical and practical reasons. In addition, the cost-effectiveness of this imaging modality was not evaluated. We consider that the additional cost of PET-CT can be largely offset by the reduction in number of futile invasive or operative procedures, which certainly need further investigation.

## Conclusion

The results of this study indicate that PET/CT is valuable in EHD detection. Abdomen PET/MRI showed comparable ability with CE-CT/CE-MRI in liver lesion detection. PET imaging had a clinical impact on 25% of patients. Hence, a simultaneous whole-body PET/CT and abdomen PET/MRI may become a new one-stop imaging method in the preoperative work-up of patients with potentially resectable colorectal liver metastases.

## Data Availability Statement

The original contributions presented in the study are included in the article/**Supplementary Material**. Further inquiries can be directed to the corresponding authors.

## Ethics Statement

The studies involving human participants were reviewed and approved by the Medical Ethics Committee of Peking University Cancer Hospital (ethical approval no. 2018KT110-GZ01). The patients/participants provided their written informed consent to participate in this study. Written informed consent was obtained from the individual(s) for the publication of any potentially identifiable images or data included in this article.

## Author Contributions

All authors listed have made a substantial, direct, and intellectual contribution to the work and approved it for publication.

## Funding

The current research was financially supported by the National Natural Science Foundation (No. 81871387), Beijing Natural Science Foundation, Jing-Jin-Ji special projects for basic research cooperation (H2018206600), Beijing Excellent Talents Funding (2017000021223ZK33), Beijing Municipal Administration of Hospitals-Yangfan Project (ZYLX201816), and Science Foundation of Peking Univesity Cancer Hospital (No. 2021-4).

## Conflict of Interest

The authors declare that the research was conducted in the absence of any commercial or financial relationships that could be construed as a potential conflict of interest.

## Publisher’s Note

All claims expressed in this article are solely those of the authors and do not necessarily represent those of their affiliated organizations, or those of the publisher, the editors and the reviewers. Any product that may be evaluated in this article, or claim that may be made by its manufacturer, is not guaranteed or endorsed by the publisher.
