# Supplementary material for: The Value of 18F-FDG PET/CT and Abdominal PET/MRI as a One-Stop Protocol in Patients With Potentially Resectable Colorectal Liver Metastases
Source: Front Oncol. 2021 Nov 9;11:714948. doi: 10.3389/fonc.2021.714948 (PMC8630637; doi:10.3389/fonc.2021.714948)
Supplement: Supplementary file 1 [file Table_1.pdf]

SI-Table 1. Acquisition parameters for the applied MRI-sequences.

Sequence	TR (msec)	TE (msec)	Matrix	FOV (mm)	Thickness (mm)	Gap (mm)	Fat Sat
WFI with trigger	5.06	2.24	256×329	350×500	4	0	NA
T2w FSE with fat saturation and trigger	4000*	88.74	320×177	380×300	6	1.2	YES
DWI (b=50,800 sec/mm^2)	4000	70	128×101	380×300	6	1.2	YES
T1w with radial acquisition	3.56	1.59	320×320	400×400	4	0	YES
Dual echo T1w with breath-hold	4.22	2.58	320×168	400×300	6	0	NA

* T2WI with fat saturation sequence uses respiratory gating, TR differs in patients due to different respiratory rate. WFI: water fat imaging; FSE: fast spin-echo
